# Language function of the superior longitudinal fasciculus in patients with arteriovenous malformation as evidenced by automatic fiber quantification

**DOI:** 10.3389/fradi.2023.1121879

**Published:** 2023-05-19

**Authors:** Fangrong Zong, Zhaoyi You, Leqing Zhou, Xiaofeng Deng

**Affiliations:** ^1^ School of Artificial Intelligence, Beijing University of Posts and Telecommunications, Beijing, China; ^2^ State Key Laboratory of Oncogenes and Related Genes, Institute for Personalized Medicine, School of Biomedical Engineering, Shanghai Jiao Tong University, Shanghai, China; ^3^ Department of Neurosurgery, Beijing Tiantan Hospital, Capital Medical University, Beijing, China; ^4^ China National Clinical Research Center for Neurological Diseases, Beijing, China

**Keywords:** diffusion tensor imaging, automatic fiber quantification, superior longitudinal fascicle, language reorganizations, arteriovenous malformation

## Abstract

The superior longitudinal fasciculus (SLF) is a major fiber tract involved in language processing and has been used to investigate language impairments and plasticity in many neurological diseases. The SLF is divided into four main branches that connect with different cortex regions, with two branches (SLF II, SLF III) being directly related to language. However, most white matter analyses consider the SLF as a single bundle, which may underestimate the relationship between these fiber bundles and language function. In this study, we investigated the differences between branches of the SLF in patients with arteriovenous malformation (AVM), which is a unique model to investigate language reorganization. We analyzed diffusion tensor imaging data of AVM patients and healthy controls to generate whole-brain fiber tractography, and then segmented the SLF into SLF II and III based on their distinctive waypoint regions. The SLF, SLF II, and III were further quantified, and four diffusion parameters of three branches were compared between the AVMs and controls. No significant diffusivity differences of the whole SLF were observed between two groups, however, the right SLF II and III in AVMs showed significant reorganization or impairment patterns as compared to the controls. Results demonstrating the need to subtracting SLF branches when studying structure-function relationship in neurological diseases that have SLF damage.

## 1. Introduction

Language plasticity has been a particular focus in neuroscience as it is important for human socialization and communication (1, 2). Patients with damages located in language-related cortical areas may have a normal language function, as has been found in patients with perinatal stroke, arteriovenous malformation (AVM), and traumatic brain injury (3–5). Among them, arteriovenous malformation is a congenital anomaly of the capillaries resulting in direct connections between cerebral arteries and veins. It occurs before the establishment of language network and is hence a unique model for investigating the mechanisms of language plasticity (6–8). Neuroimaging methods such as functional magnetic resonance imaging (MRI) and electrocorticography (ECoG), neuropsychology, and psycholinguistics have been applied to study language plasticity (9, 10). However, it was found that although language-related cortical regions defined by fMRI are activated by speech tasks, they may not be essential for speech execution. An electroencephalographic (EEG) study showed that cortical sites essential for language were distributed more discretely than the ones identified by fMRI under similar tasks, indicating varied patterns of recovery and reorganization (11). This attributes to the fact that our language network in AVM is rather complex.

Historically, syndrome-related brain regions were described and generalized into language models. In the “Broca-Wernicke-Geschwind Model” (12), the inferior frontal gyrus is described as an expressive language center and the posterior temporal cortex, as the receptive language center. Hickok and Poppel further proposed a “dual-stream model” for language analogous to the cortical organization of vision (13). In this model, the sensory representation interfaced with conceptual representation via projections to the temporal lobe, which is defined as the ventral stream, and with motor representation via projections to temporal-parietal regions, which is described as the dorsal stream. The dorsal stream functions mainly on the dominant hemisphere, while the ventral stream is likely activated bilaterally (14). In these two streams, white matters connect different language centers, and therefore the microstructure of white matters are closely related to language function (15). For instance, the dorsal stream is mainly composed of arcuate fascicle (AF) and superior longitudinal fascicle (SLF). In the ventral stream, uncinate fascicles (UNC) and inferior fronto-occipital fascicle (IFOF) are the two main pathways, with inferior longitudinal fascicle (ILF) and middle longitudinal fascicle as assistance.

Diffusion tensor imaging (DTI) is a unique method to detect microstructural changes, which acquires the signal sensitive to diffusion movement of water molecules in the brain's white matter (16, 17). DTI reveals both the extent of diffusion anisotropy and the predominant direction of water diffusion in image voxels, offering a non-invasive method to study the fiber structures in the brain (18, 19). The relationship between white matter fibers and language function have been studied over past decades. For instance, the left ILF and left AF were found to be positively correlated with language function in healthy participants. In a larger cohort study, IFOF, SLF and UNC are found to be closely related to language function (20). In a previous AVM study (21), remodeling of the left SLF was found in patients with frontal lesions by using an automatic fiber quantification (AFQ) procedure (22), with no evidence of such mechanism in patients with parietal and temporal lesions. Moreover, no significant differences were seen in the right SLF.

SLF describes a group of longitudinally oriented, associative fibers that connect the frontal lobes with the temporal lobes and parietal lobes of the brain (23, 24). From super to inferior and from medial to lateral directions, the SLF is divided into three main subcomponents: SLF I, SLF II, and SLF III. Each subcomponent plays a different role in language processing. SLF I (most superior and medial), SLF II (having an intermediate course), SLF III (most inferior and lateral), and SLF-tp (inferior temporal to posterior temporal cortex). However, most of the white matter analyses considered SLF as a single bundle, the relationship between these subcomponents and language function were not investigated in detail. By using a two-tensor Unscented Kalman Filter (UKF) method (25), branches of SLF (SLF II and SLF III) were segmented and analyzed in healthy participants. The number of streamlines of the left SLF-III and right SLF-III were found to influence semantic memory performance and emotion perception performance, respectively. How SLF-II and III interact with language reorganization in AVM patients also needs further investigation.

In this study, we categorized patients according to their fiber impairments and studied the diffusion properties in more detail. Additionally, SLF II and SLF III were automatically segmented and studied. The SLF-I definition is debated in the literature and SLF-tp is usually described as a component of the arcuate fasciculus (AF) (26), therefore these two branches were excluded in this study. Since the UKF method requires multiple non-zero diffusion sensitizing gradients which is usually not possible in clinical scanner, we used the AFQ toolbox (22) to process the acquired datasets. The connectivity was estimated and compared with the controls at different locations on the fiber tract. We attempted to attain a common pattern of language reorganization in AVM patients that might be a good model for research on language development and language plasticity.

## 2. Methods and materials

### 2.1. Participants and language function assessment

This is a retrospective study, and 32 AVM patients aged 18–60 years were included. All participants were native Mandarin Chinese speakers and righthanded, confirmed by the Edinburgh handedness inventory. All patients had AVM lesions in the left hemisphere, in other words, exclusively the language dominant hemisphere. The lesion regions included language-related areas such as the middle and inferior frontal gyrus, precentral gyrus, superior and middle temporal gyrus, supra-marginal gyrus, and angular gyrus. This study was approved by the Institutional Review Board of Beijing Tiantan Hospital, Capital Medical University (number: KY2018-103-01), and written informed consent was obtained from all participants the study was registered in the Chinese Trial Registry (clinical trial number: ChiCTR1900020993).

Lesions at different locations may lead to varied patterns of language reorganization due to different pathological mechanism (27). Thus, lesion location, serving as a major factor for language plasticity, was taken into account in this study. The enrolled 32 patients were reassigned depending on their affected fiber tracts to investigate the associations between fiber damage and its microstructural changes.

To identify the location where fibers are affected by the lesions, we compared the AVM lesions map with the fiber probabilistic map (28). Patients with lesions in the fiber area at a probability >0.5 were selected for statistical analysis, but patients with multiple sporadic damages or <50% possibility of having lesions in the fiber areas were excluded.

The Western Aphasia Battery (WAB) is widely used to diagnose aphasia and assess language disturbances in patients with brain lesions. The Chinese version of the WAB was administered to all participants to assess their language function. Four subtests were included: fluency, comprehension, repetition, and naming. Aphasia severities were estimated in the Aphasia quotients. The score of the four subtests can be used to classify the type of aphasia, because it estimates the integrity of cortical function in language (29).

### 2.2. Image acquisition and data processing

Magnetic resonance imaging experiments were performed on a 3 T MRI scanner with a 20-element head-neck coil. The imaging parameters and data preprocessing for both DTI and T1-weighted imaging were reported in our previous study (21). In specific, a T1-weighted magnetization-prepared rapid-acquisition gradient-echo sequence was applied by using following parameters: repetition time (TR) = 2,530 ms, echo time (TE) = 3.37 ms, flip angle = 7°, field of view = 256 mm × 256 mm, matrix = 256 × 256, voxel size = 1.0 mm × 1.0 mm × 1.0 mm, and number of slices = 176. A spin-echo (SE) echo planar imaging (EPI) was acquired for the diffusion dataset by using following parameters: TR = 8,100 ms, TE = 75 ms, matrix = 100 × 100 × 72, voxel size = 1.5 mm × 1.5 mm × 1.5 mm. In addition to 3 frames with *b* = 0 s/mm^2^, we acquired 64 frames along isotropically-distributed diffusion directions with *b* = 1,000 s/mm^2^.

The DTI data of subjects underwent a preprocessing pipeline including eddy current, subject movement and field bias corrections followed by automatic fiber tracking with the AFQ method (22). The fiber assignment by continuous tracking (FACT) arithmetic was applied for deterministic tractography of the whole brain (18). The termination rule for tracing was when the FA value of the voxel was less than 0.2 or the minimum angle between the last path segment and next step was greater than 30 degrees. The result was then classified into 20 major fiber tracts (including the whole SLF tract) in the brain by using the waypoint region of interests (ROIs) method (30). The waypoint ROIs integrated in the AFQ toolbox were registered from the MNI template space into individual coordinate space via a non-linear transformation. Fibers pass through same waypoint ROI pairs were categorized as one tract. By comparing fibers in one tract with the fiber tract probability maps that were previously warped into each individual space, the fibers were scored according to their probability values. Fibers that had lowest probability score or their lengths were far away from the center would be discarded.

### 2.3. SLF segmentation

**Figure 1:**
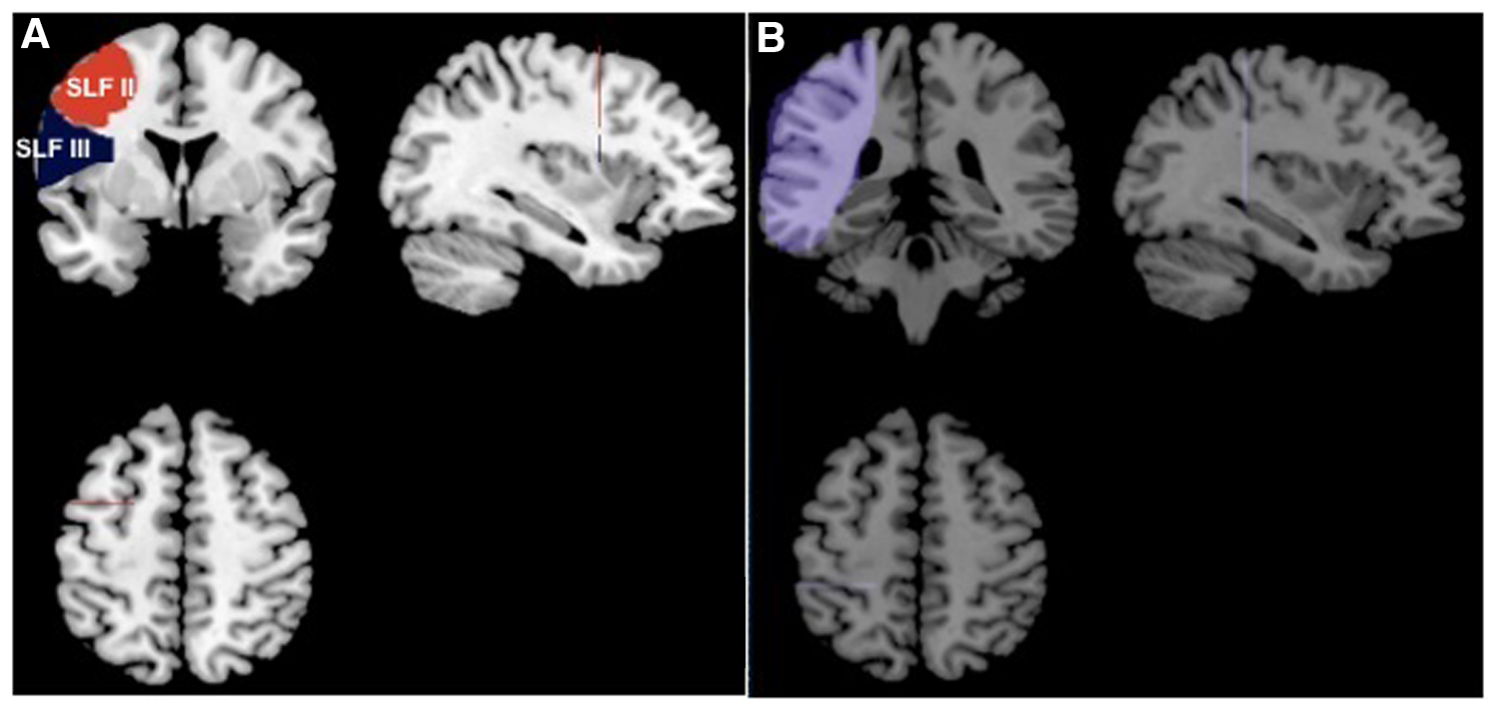
ROIs for segmentation of SLF-II and SLF-III. (**A**) Two ROIs defined on the coronal slice at MNI coordinate *y* = 2. The ROI in red is for SLF-II and the blue one is for SLF-III; (**B**) the ROI for both SLF-II and SLF-III was defined on the coronal slice at MNI coordinate *y* = −25.

To analyze the subcomponents of SLF, fibers in SLF need to be further segmented. Based on the axonal tracing results of monkey brains, *in vivo* dissection of SLF was adapted to human brains (31, 32): two different ROIs (Figure 1A) are defined on the same coronal slice at the anterior commissure (AC). A common ROI (Figure 1B) was drawn on the coronal slice at the posterior commissure (PC). All ROIs in this study were drawn in the standard Montreal Neurological Institute (MNI) space (33). The ROIs were then transformed into individual native space. These ROIs were integrated in the AFQ toolbox (http://yeatmanlab.github.io/pyAFQ/) on Matlab 2012a (Mathwork, USA), to allow for an automatic quantification of SLF branches in all participants. SLF II and III were then segmented and refined by using the waypoint ROIs in Figures 1A,B. Fibers that passed through the SLF II or III waypoint ROI pair belonged to the corresponding SLF subbranches. Subsequently, SLF, SLF II and SLF III were resampled to 100 nodes that were equally spaced along the fiber tract. Diffusion properties were calculated in each node: fractional anisotropy (FA), mean diffusivity (MD), axial diffusivity (AD), and radial diffusivity (RD).

### 2.4. Statistical analysis

Independent sample *t*-test were used to estimate the significant differences in FA, MD, AD, and RD values between AVM and control participants. The *p*-value after family-wise error (FWE) correction was used for multiple comparison of 100 nodes to reduce the occurrence of false positive results. The *p*-value *p* < 0.05 were deemed to be significant.

## 3. Results

### 3.1. Participant characteristics

By comparing the AVM lesions map with the fiber probabilistic map, 8 patients were found to have lesions in IFOF or ILF (ventricle stream) and 7 were found to have deficits in SLF or AF (dorsal stream). We attempted to separate them into two AVM groups (ventricle damage group and dorsal damage group). However, after performing the statistical power analysis (34), we found that minimal 12 subjects are required to reach a power effect of 0.8. Therefore, these 15 patients were selected and grouped as one AVM group. As a result, 15 age- and sex-matched healthy controls were included in the statistical analysis.

The result of Pearson's chi-squared test on sex and Student's *t*-test on age showed that subjects in the AVM group were well-matched with the control group regarding the age and sex distribution. All the patients had normal language function according to the WAB results shown in our previous study (21).

**Figure 2:**
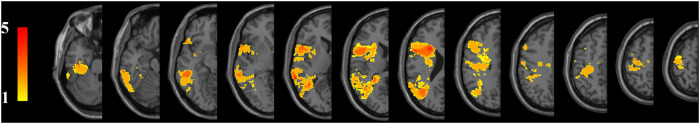
Overlapping lesions in these AVM participants. Twelve axial slices of the left hemisphere are shown from MNI coordinate *z* = −27 to *z* = 64. The colored intensity indicates the number of lesions in this region.

Overlapping lesions for the selected 15 patients were drawn in the standard MNI space and visualized in Figure 2. Ten coronal slices at the MNI z-coordinate from −27 to 64 were chosen to be shown. The color indicates the number of patients that have lesions in the covered region. As can be seen from Figure 2, AVM lesions involves the frontal, parietal, and temporal lobes, with the SLF being the most affected fiber tracts.

### 3.2. Tractography and quantification

**Figure 3:**
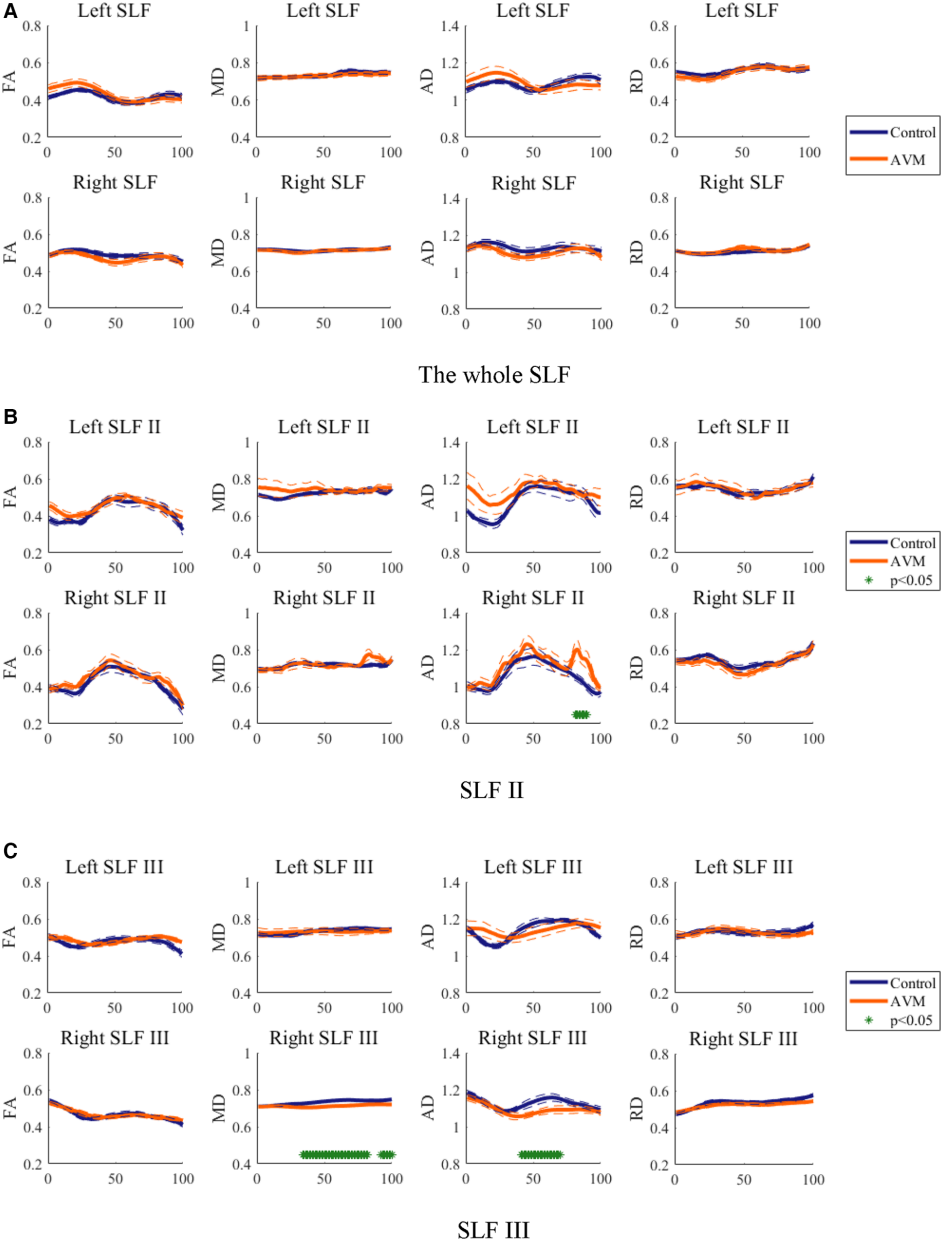
Diffusion metrics of FA, AD, and RD on each node of (**A**) SLF, (**B**) SLFII and (**C**) SLF III in both hemispheres. Blue and red curves stand for the AVM and control groups, respectively. *Represents the *p* value is less than 0.05 and deemed to be significant in this study.

Diffusion properties was traditionally averaged over an entire fiber tract for analysis, whilst FA value varies systematically along the fiber tract (16). To mitigate the intra-tract difference in this study, each tract was cut into 100 nodes and the diffusion value was compared between patients and controls at each node. Diffusion metrics including FA, AD, and RD were measured on each node of the SLF (Figure 3A). Diffusion property profiles were drawn in a solid line for the mean value parallel with two dash lines for standard error. The significant difference is marked in asterisks.

The FA, RD, and AD profiles of the SLF of patients are similar to the controls but vary at specific locations along tracts. Thus, it is reasonable to compare the diffusion properties of patients with control ones along fiber tracts. The FA, MD, AD and RD of the whole right SLF tractography showed no significant difference between AVM patients and healthy controls.

### 3.3. SLF segmentation and analysis

SLF-II and SLF-III were extracted from the whole SLF tractography, and the quantifications were visualized in Figures 3B,C. Mean FA, MD, AD and RD values of the three SLFs in two groups in more than 10 nodes are summarized in Table 1. This definition has been used in the classification of Alzheimer's disease using multisite DTI datasets (35). Values of the whole SLF is also summarized in Table 1 for better comparison.

**Table 1:** Mean FA, AD, RD and MD values in different SLF branches.

Groups	Fascicles	FA	MD	AD	RD
AVM	Left SLF	0.43 ± 0.08	0.73 ± 0.04	1.1 ± 0.11	0.55 ± 0.06
	Left SLF II	0.45 ± 0.09	0.74 ± 0.09	1.12 ± 0.15	0.54 ± 0.1
	Left SLF III	0.49 ± 0.05	0.73 ± 0.05	1.14 ± 0.1	0.52 ± 0.05
	Right SLF	0.47 ± 0.06	0.71 ± 0.02	1.11 ± 0.07	0.51 ± 0.04
	Right SLFII	0.44 ± 0.1	0.72 ± 0.07	1.1 ± 0.15**	0.53 ± 0.08
	Right SLFIII	0.47 ± 0.05	0.71 ± 0.03**	1.09 ± 0.07**	0.52 ± 0.04
Control	Left SLF	0.42 ± 0.06	0.73 ± 0.03	1.08 ± 0.07	0.56 ± 0.05
	Left SLF II	0.44 ± 0.1	0.73 ± 0.04	1.09 ± 0.13	0.54 ± 0.06
	Left SLF III	0.47 ± 0.06	0.73 ± 0.03	1.14 ± 0.08	0.53 ± 0.05
	Right SLF	0.48 ± 0.06	0.72 ± 0.03	1.12 ± 0.08	0.52 ± 0.04
	Right SLFII	0.44 ± 0.11	0.72 ± 0.05	1.09 ± 0.17	0.54 ± 0.06
	Right SLFIII	0.47 ± 0.05	0.74 ± 0.03	1.13 ± 0.08	0.54 ± 0.04

** Indicates *p* < 0.05.

As can be seen in Table 1 and Figure 3, there were different changing patterns of SLF-II and SLF-III compared with the whole SLF. The FA and RD values of left and right SLF II and III were slightly different in the AVM group, but not significantly compared to the control group. No obvious MD differences of left SLF II and III in two groups, except that right SLF III had a statistically lower value in the AVM group. Similarly, the AD value of the right SLF III in the AVM group was lower than that in the control group. However, a statistically high AD value of the right SLF II were found in AVM, whereas no AD differences of left SLF, SLF II or III between two groups. In addition, more fluctuations of these diffusivity parameters were observed along right SLF II than SLF III. It is possible that the two SLF subbranches of AVM patients have different mechanism in language function. It is noted that the FA, MD, AD and RD values of SLF were not simply the mean value of SLF II and III due to the effect of SLF I which were irrelevant to language function.

## 4. Discussion

In this study, patients with AVM were reassigned into one group according to lesion locations on language related white matters. Thus, the interaction between damaged fiber tracts and white matter with significant changes could be revealed. Importantly, significant changes were only found in the right hemisphere, which might indicate the relatively dominant role that the right hemisphere plays in language function of AVM patients. We found no obvious differences of the left SLFs between the AVM and control groups. An enhanced pattern in right SLF II but impairment patterns in right SLF III when comparing quantitative diffusivity values were seen in two groups. All patients have normal language function, leading to the fact that a language reorganization may occur in the right hemisphere of AVM patients.

Different patterns of diffusion properties’ changes were reported in different clinical cases. The FA value, defined as a normalized standard deviation of eigenvalues, is commonly referred to as a summary of microstructure integrity. The MD value measures the membrane density and fluid viscosity and is rather insensitive to fiber changes. The RD and AD values are the apparent diffusivity in the direction perpendicular and parallel to the fibers, respectively. RD is sensitive to changes in myelin and increases during demyelination. AD is variable to the change of axon and increases with brain maturation (36, 37). In this study, only significant differences were seen in MD and AD, possibly due to small sample sizes included in both groups.

SLF plays an important role in language processing. Specifically, parts of the SLF have existed since birth, which connects the temporal cortex to the premotor cortex in the frontal lobe. While, the other part develops later after birth which connects the temporal cortex to the Broca's cortex (23, 24). Thus, it is thought that these AVM lesions may affect the formation of the left SLF. However, no significant differences on the left SLF between two groups were found. The whole bilateral SLF tractography showed no significant difference between the patients and controls. These results were consistent with the previous study (21), indicating a more complex mechanism of the formation of SLF bilaterally along with the development of AVM lesions.

After resampling SLF II and SLF III from the whole brain tractography, significant differences were found in the SLF II of the right hemisphere in the AVM group. The higher FA value in the left SLF II, lower RD value in the left and right SLF II may intensify the white matter integrity and myelination of SLF II in both hemispheres. SLF II occupies the central core of the white matter above the insula. It extends from the angular gyrus to the dorsolateral prefrontal and premotor cortex. SLF III locates ventrally to SLF II, in the parietal and frontal opercula. It connects the supramarginal gyrus with the ventral premotor and prefrontal regions. SLF III was found to be the crucial fiber tract in articulation, because patients with electrical stimulation on SLF III have dysarthria and anarthria (38). However, neural reorganization in SLF II was found to be more prominent than in SLF III leading to a specific function in language processing. Nevertheless, we found no significant diffusivity differences of the SLF II and III between the AVM and control groups. As can be seen from Figure 2, the left SLFs were largely affected by the lesions, that may lead to a complex formation of these subbranches, eliminating inter-group differences.

It should be mentioned that patients were grouped with lesions damaging language-related tracts in this study, including SLF, IFOF, AF and ILF. They were previously gathered with lesions in a specific cortical region in another study (21). We aimed to mitigate the inter-correlating effects of different cortex regions but linked through same white matters. However, such a delicate grouping method excludes more than half the patients in our previous study, resulting in a small sample size. Confined by clinical cases, AVM is a con disease with relatively small population as compared to stroke or glioma. Small numbers of patients possibly reduced the validity of the results. Futural work could be anticipated to include more AVM patients and group them according to individual damaged fiber tract.

Besides, this study has limitations. The number of fibers of extracted SLF-II was rather small compared with the SLF-III. More advanced acquisition methods, such as High angular resolution diffusion imaging (39) and DSI (40), and analytical models, such as constrained spherical deconvolution (41) and UKF (25), need to be promoted in clinical studies and utilized to overcome the crossing fiber constraints. In this way, more quantitative parameters such as fiber density and shape could be compared between groups to explore the underlying mechanisms of SLF and other language-related tracts.

## 5. Conclusion

To the best of our knowledge, this is the first study to investigate the language function of SLF branches in AVM patients. We redefined the ROIs of the SLF branches and integrated in the AFQ toolbox for the automatic isolation of subcomponents. The diffusion matrices at each node on the two main branches of SLF were subsequently calculated and analyzed. Language plasticity of right SLF II were found in the AVM groups as compared to the healthy controls. The detailed information about the microstructure of SLF II, III and whole SLF offers the possibility to investigate the structure-function relationship of other neurological diseases, especially the function depending on three SLF subcomponents such as emotion, and memory.

## Data Availability

The raw data supporting the conclusions of this article will be made available by the authors, without undue reservation.

## References

[1] Neville HJ Bavelier D. Neural organization and plasticity of language. Curr Opin Neurobiol. (1998) 8:254–8. 10.1016/S0959-4388(98)80148-7 9635210

[2] Martin KC Ketchabaw WT Turkeltaub PE. Plasticity of the language system in children and adults. Handb Clin Neurol. (2022) 184:397–414. 10.1016/B978-0-12-819410-2.00021-7 35034751 PMC10149040

[3] Newport EL Seydell-Greenwald A Landau B Turkeltaub PE Chambers CE Martin KC Language and developmental plasticity after perinatal stroke. Proc Natl Acad Sci. (2022) 119:e2207293119. 10.1073/pnas.2207293119 36215488 PMC9586296

[4] Lazar RM Marshall RS Pile-Spellman J Duong HC Mohr JP Young WL Interhemispheric transfer of language in patients with left frontal cerebral arteriovenous malformation. Neuropsychologia. (2000) 38:1325–32. 10.1016/S0028-3932(00)00054-3 10869575

[5] Vicari S Albertoni A Chilosi AM Cipriani P Cioni G Bates E Plasticity and reorganization during language development in children with early brain injury. Cortex. (2000) 36(1):31–46. 10.1016/S0010-9452(08)70834-7 10728895

[6] Chen CJ Ding D Derdeyn CP Lanzino G Friedlander RM Southerland AM Brain arteriovenous malformations: a review of natural history, pathobiology, and interventions. Neurology. (2020) 95:917–27. 10.1212/WNL.0000000000010968 33004601

[7] Fleetwood IG Steinberg GK. Arteriovenous malformations. Lancet. (2002) 359:863–73. 10.1016/S0140-6736(02)07946-1 11897302

[8] Solomon RA Connolly ES Jr. Arteriovenous malformations of the brain. N Engl J Med. (2017) 376:1859–66. 10.1056/NEJMra1607407 28489992

[9] Tzourio-Mazoyer N Perrone-Bertolotti M Jobard G Mazoyer B Baciu M. Multi-factorial modulation of hemispheric specialization and plasticity for language in healthy and pathological conditions: a review. Cortex. (2017) 86:314–39. 10.1016/j.cortex.2016.05.013 27321148

[10] Deng X Wang B Zong F Yin H Yu S Zhang D Right-hemispheric language reorganization in patients with brain arteriovenous malformations: a functional magnetic resonance imaging study. Hum Brain Mapp. (2021) 42(18):6014–27. 10.1002/hbm.25666 34582074 PMC8596961

[11] Chang EF Raygor KP Berger MS. Contemporary model of language organization: an overview for neurosurgeons. J Neurosurg. (2015) 122(2):250–61. 10.3171/2014.10.JNS132647 25423277

[12] Dick AS Tremblay P. Beyond the arcuate fasciculus: consensus and controversy in the connectional anatomy of language. Brain. (2012) 135(12):3529–50. 10.1093/brain/aws222 23107648

[13] Hickok G Poeppel D. The cortical organization of speech processing. Nat Rev Neurosci. (2007) 8(5):393–402. 10.1038/nrn2113 17431404

[14] Hickok G Poeppel D. Dorsal and ventral streams: a framework for understanding aspects of the functional anatomy of language. Cognition. (2004) 92(1–2):67–99. 10.1016/j.cognition.2003.10.011 15037127

[15] Bernal B Altman N. The connectivity of the superior longitudinal fasciculus: a tractography DTI study. Magn Reson Imaging. (2010) 28(2):217–25. 10.1016/j.mri.2009.07.008 19695825

[16] Basser PJ Mattiello J LeBihan D. MR diffusion tensor spectroscopy and imaging. Biophys J. (1994) 66(1):259–67. 10.1016/S0006-3495(94)80775-1 8130344 PMC1275686

[17] Alexander AL Lee JE Lazar M Field AS Diffusion tensor imaging of the brain. Neurotherapeutics. (2007) 4(3):316–29. 10.1016/j.nurt.2007.05.011 17599699 PMC2041910

[18] Mori S Vanzijl PC. Fiber tracking: principles and strategies a technical review. NMR Biomed. (2002) 15(7–8):468–80. 10.1002/nbm.781 12489096

[19] Friederici AD. White-matter pathways for speech and language processing. Handb Clin Neurol. (2015) 129:177–86. 10.1016/B978-0-444-62630-1.00010-X 25726269

[20] Zekelman LR Zhang F Makris N He J Chen Y Xue T White matter association tracts underlying language and theory of mind: an investigation of 809 brains from the human connectome project. Neuroimage. (2022) 246:118739. 10.1016/j.neuroimage.2021.118739 34856375 PMC8862285

[21] Deng X Yin H Zhang Y Zhang D Wang S Cao Y Impairment and plasticity of language related white matter in patients with brain arteriovenous malformations. Stroke. (2022) 53(5):1682–91. 10.1161/STROKEAHA.121.035506 34847706

[22] Yeatman JD Dougherty RF Myall NJ Wandell BA Feldman HM Tract profiles of white matter properties: automating fiber tract quantification. PLoS One. (2012) 7(11):e49790. 10.1371/journal.pone.0049790 23166771 PMC3498174

[23] Schmahmann JD Smith EE Eichler FS Filley CM. Cerebral white matter: neuroanatomy, clinical neurology, and neurobehavioral correlates. Ann N Y Acad Sci. (2008) 1142:266–309. 10.1196/annals.1444.017 18990132 PMC3753195

[24] Martino J DeWittHamer PC Berger MS Lawton MT Arnold CM de Lucas EM Analysis of the subcomponents and cortical terminations of the perisylvian superior longitudinal fasciculus: a fiber dissection and DTI tractography study. Brain Struct Funct. (2013) 218(1):105–21. 10.1007/s00429-012-0386-5 22422148

[25] Malcolm JG Shenton ME Rathi Y. Filtered multitensor tractography. IEEE Trans Med Imaging. (2010) 29(9):1664–75. 10.1109/TMI.2010.2048121 20805043 PMC3045040

[26] Makris N Kennedy DN McInerney S Sorensen AG Wang R Caviness Jr VS Segmentation of subcomponents within the superior longitudinal fascicle in humans: a quantitative, in vivo, DT-MRI study. Cereb Cortex. (2005) 15(6):854–69. 10.1093/cercor/bhh186 15590909

[27] Huang L Chen X Sun W Chen H Ye Q Yang D Early segmental white matter fascicle microstructural damage predicts the corresponding cognitive domain impairment in cerebral small vessel disease patients by automated fiber quantification. Front Aging Neurosci. (2021) 12:598242. 10.3389/fnagi.2020.598242 33505302 PMC7829360

[28] Kubicki M McCarley R Westin CF Park HJ Maier S Kikinis R A review of diffusion tensor imaging studies in schizophrenia. J Psychiatr Res. (2007) 41(1–2):15–30. 10.1016/j.jpsychires.2005.05.005 16023676 PMC2768134

[29] Shewan CM Kertesz A. Reliability and validity characteristics of the western aphasia battery (WAB). J Speech Hear Disord. (1980) 45(3):308–24. 10.1044/jshd.4503.308 7412225

[30] Wakana S Caprihan A Panzenboeck MM Fallon JH Perry M Gollub RL Reproducibility of quantitative tractography methods applied to cerebral white matter. Neuroimage. (2007) 36(3):630–44. 10.1016/j.neuroimage.2007.02.049 17481925 PMC2350213

[31] de Schotten MT Dell'Acqua F Valabregue R Catani M. Monkey to human comparative anatomy of the frontal lobe association tracts. Cortex. (2012) 48(1):82–96. 10.1016/j.cortex.2011.10.001 22088488

[32] Fitzgerald J Leemans A Kehoe E O'Hanlon E Gallagher L McGrath J. Abnormal fronto-parietal white matter organisation in the superior longitudinal fasciculus branches in autism spectrum disorders. Eur J Neurosci. (2018) 47(6):652–61. 10.1111/ejn.13655 28741714

[33] Fonov V Evans AC Botteron K Almli CR McKinstry RC Collins DL. Brain development cooperative group. Unbiased average age-appropriate atlases for pediatric studies. Neuroimage. (2011) 54(1):313–27. 10.1016/j.neuroimage.2010.07.033 20656036 PMC2962759

[34] Chow SC Shao J Wang H Lokhnygina Y. Sample size calculations in clinical research. New York: CRC Press (2017).

[35] Qu Y Wang P Liu B Song C Wang D Yang H AI4AD: artificial intelligence analysis for Alzheimer's disease classification based on a multisite DTI database. Brain Disorders. (2021) 1:100005. 10.1016/j.dscb.2021.100005

[36] Simon NG Lagopoulos J Paling S Pfluger C Park SB Howells J Peripheral nerve diffusion tensor imaging as a measure of disease progression in ALS. J Neurol. (2017) 264(5):882–90. 10.1007/s00415-017-8443-x 28265751

[37] Winklewski PJ Sabisz A Naumczyk P Jodzio K Szurowska E Szarmach A Understanding the physiopathology behind axial and radial diffusivity changes what do we know? Front Neurol. (2018) 9:92. 10.3389/fneur.2018.00092 29535676 PMC5835085

[38] Maldonado IL Moritz-Gasser S Duffau H. Does the left superior longitudinal fascicle subserve language semantics? A brain electrostimulation study. Brain Struct Funct. (2011) 216:263–74. 10.1007/s00429-011-0309-x 21538022

[39] Descoteaux M. High angular resolution diffusion imaging (HARDI). Wiley Encyc Electric Electron Eng. (1999) 27:1–25. 10.1002/047134608X.W8258

[40] Wedeen VJ Wang RP Schmahmann JD Benner T Tseng WY Dai G Diffusion spectrum magnetic resonance imaging (DSI) tractography of crossing fibers. Neuroimage. (2008) 41(4):1267–77. 10.1016/j.neuroimage.2008.03.036 18495497

[41] Tournier JD Calamante F Connelly A. Robust determination of the fibre orientation distribution in diffusion MRI: non-negativity constrained super-resolved spherical deconvolution. NeuroImage. (2007) 35:1459–72. 10.1016/j.neuroimage.2007.02.016 17379540

